# Investigating Consumer Attitude and Intention towards Online Food Purchasing in an Emerging Economy: An Extended TAM Approach

**DOI:** 10.3390/foods8110576

**Published:** 2019-11-15

**Authors:** Thi Thu Ha Nguyen, Ninh Nguyen, Thi Bich Loan Nguyen, Thi Thu Hoai Phan, Lan Phuong Bui, Hee Cheol Moon

**Affiliations:** 1 Department of External Affairs and Communications, Thuongmai University, Hanoi 100000, Vietnam; 2 Department of Entrepreneurship, Innovation and Marketing, La Trobe Business School, La Trobe University, Bundoora, VIC 3086, Australia; 3 Business Sustainability Research Group; Thuongmai University, Hanoi 100000, Vietnam; 4 Faculty of Business Administration, Thuongmai University, Hanoi 100000, Vietnam; bichloandhtm@tmu.edu.vn; 5 Faculty of Marketing, Thuongmai University, Hanoi 100000, Vietnam; hoaiphan@tmu.edu.vn (T.T.H.P.); builanphuong@tmu.edu.vn (L.P.B.); 6 Department of International Trade, Chungnam National University, Daejeon 34134, Korea; hcmoon@cnu.ac.kr

**Keywords:** online food shopping, technology acceptance model, website, trust, attitude, purchase intention, emerging economy, Vietnam

## Abstract

Along with the development of the Internet and technology, food retailers have increasingly adopted online channels that enable consumers to buy food products online. This research aims to investigate the factors that influence consumer attitude and intention towards online food purchasing. A research framework was developed by combining the technology acceptance model with website trust, which is an important facilitator of online shopping. Using an online survey, data were obtained from 319 online food shoppers in an Asian emerging economy, i.e., Vietnam. Results from structural equation modeling show that perceived usefulness, perceived ease of use, and website trust are important drivers of attitude towards online food purchasing. Among these drivers, perceived ease of use has the greatest impact on attitude. Additionally, attitude and website trust exert a direct and positive effect on intention towards online food purchasing. Taken together, these findings have important managerial implications for key stakeholders, such as online food retailers, associations, and policy makers. One key implication is that online food sellers must endeavor to make their websites simple to use, easy to navigate, reliable, and secure. Several potential caveats for future research studies are also presented in this paper.

## 1. Introduction

The Internet and mobile technology have significant impacts on consumers and businesses. According to Nielsen [1], there are 4 billion people connected to the internet, and 92.6% of them use mobile devices for internet access. Consumers have increasingly spent more time on a diverse range of digital activities, with greater frequency [1,2]. Online shopping is a dramatically developing business, since internet technology and applications provide customers with more accessible, more convenient, and cheaper methods to find more varied kinds of products than traditional shopping [3,4]. Along with the growth and associated advantages of online shopping, purchasing food online is climbing at an unprecedented rate [2,5], and generation X, Millennials, and generation Z are the most avid online food buyers [6].

Vietnam is recognized as one of the nations with the fastest rates of internet penetration (ranked 17th in the world) and increasing smart phone penetration rates [7], offering great potential for the development of online shopping. The latest data from Nielsen [1] demonstrates that 98% of the Vietnamese who have access to the Internet have made at least one purchase online, growing 1% compared to 2017. The report also reveals that 17% of digital consumers have shopped for fresh food through online channels, which is up 5% compared to 2017. In general, consumers can order and purchase food products from a diverse range of websites for grocery stores, supermarkets, restaurants, food intermediaries, and delivery businesses, such as Vietnammm.com, Now.vn (Delivery Now), and Flyfood.vn. It is estimated that Vietnamese consumers are likely to shop for more food products online in 2019 than those consumers in other Southeast Asian markets [8]. Given that many online shoppers visit virtue stores on social network platforms [9], this inevitable trend has been speeded up by a dramatic increase in the number of social media users (e.g., Facebook) in the country [5,10].

Although the aforementioned figures show that there has been a rapid expansion of online food shopping in Vietnam, research on the determinants of consumer attitude and behavior associated with online food purchasing remains limited. A notable study conducted by Kim Dang et al. [10] revealed that shoppers in Hanoi, which is the capital of Vietnam, tend to purchase food online because of competitive price, convenience, and food safety. However, this study did not comprehensively investigate the effects of technology acceptance and trust in online vendors on consumer attitude and intention towards online food purchases. According to Van der Heijden et al. [11], research on online consumer behavior should investigate two types of issues differentiating online consumers from traditional consumers, i.e., technology interaction and trust. This assertion is also supported by several authors [12,13,14]. Additionally, a comprehensive review by Changchit et al. [15] demonstrates that the technology acceptance model (TAM) by Davis et al. [16] has been widely applied in prior studies which investigated internet shopping behavior. However, given that such studies primarily concentrate on developed countries and markets, their findings may not be applicable to emerging economies [15].

The aim of this paper is to investigate the factors that influence consumer attitude and intention towards purchasing food online in an emerging country. The study extends the TAM by combining its key constructs with trust, which appears to be a key determinant of online shopping behavior [11,12,17,18,19,20]. It therefore adds to the ongoing debate regarding the antecedents and determinants of online food purchasing. By focusing on Vietnam, this study enriches the extant knowledge on technology acceptance and online food purchases in developing and emerging market economies. Practically, it offers fresh insights into how different factors enhance online consumers’ purchases of food products in the emerging Asian market of Vietnam. Essentially, this would assist key stakeholders, such as online food retailers, associations, and policy makers, to develop and manage their strategies and initiatives, which aim to promote the purchase of online food products.

The remainder of this research is structured as follows. Initially, we discuss the theoretical background for this research and develop the research hypotheses. This is followed by the research methodology, which includes the questionnaire, measures, data collection, and sample. Next, we present the data analysis and results. Thereafter, a detailed discussion of the key findings and their implications are provided. Finally, we present the concluding remarks, research limitations, and directions for future research to address such limitations.

## 2. Theoretical Background and Hypotheses

### 2.1. Theoretical Background

**Figure 1:**
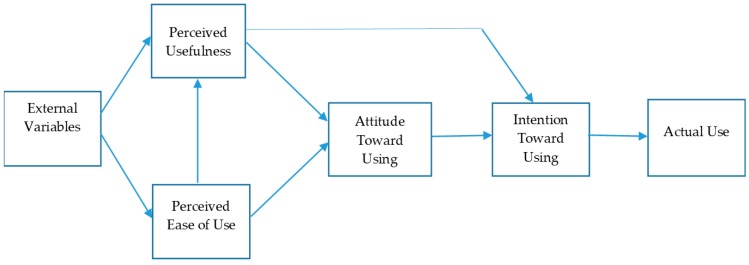
The original technology acceptance model (TAM). (*Source:* Davis et al. [16].)

Davis [21] first proposed TAM in 1986 to theorize user behavior towards computer technology, and this model was based on the Theory of Reasoned Action (TRA) postulated by Fishbein and Ajzen [22]. Essentially, the TRA has been widely applied in the areas of social psychology and marketing, and it postulates that a person’s behavior is determined by his or her intention. Intention, in its turn, is jointly decided by his or her attitude and subjective norm. Davis and his colleagues [16,23] further developed TAM into an influential research model in the information system domain by emphasizing the cognitive and affective determinants of technology acceptance. Specifically, the TAM postulates that perceived ease of use (PEOU) and perceived usefulness (PU) together determine attitude, which in turn leads to intention to use a new system or technology. It also posits that such an intention is the best predictor of the actual system use. TAM has proved to be applicable to explaining consumer attitude, intention, and behavior in various contexts and areas, such as e-learning, internet and mobile banking, internet and website usage, and e-commerce [24]. Figure 1 depicts the original TAM [16].

Many studies in the context of online shopping have drawn on the TAM [25,26,27]. Gefen et al. [26] suggest that a website is an information technology; hence, the TAM should be viable to explain consumer intention to purchase online. This argument is supported by empirical findings showing that TAM is an effective theoretical framework that successfully predicts and explains the adoption of e-commerce, including consumer behavioral intention and actual behaviors towards purchasing online [14,15,27,28,29,30]. Therefore, our research framework is underpinned by the TAM suggested by Davis et al. [16]. In addition, trust in online food retailers’ websites (i.e., website trust) is incorporated into our framework to better explain consumer attitudes and intentions related to purchasing food online. In general, trust is crucial in e-commerce [26], and it is essential to the success of online grocery retailers [31,32]. Furthermore, such a construct is particularly relevant to understanding online food purchasing decisions in an emerging market like Vietnam. Whilst Vietnamese consumers pay a lot of attention to food-borne diseases, food poisoning, and food safety issues [33,34], shopping for food online means that they are unable to see, touch, or taste the food products. Additionally, cyber activities present serious threats to personal privacy and security when making online transactions.

**Figure 2:**
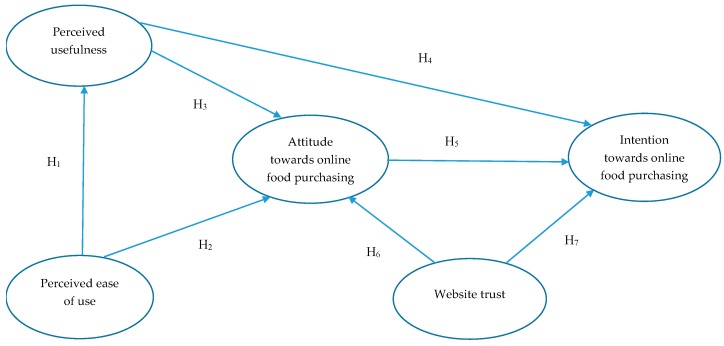
Proposed research model.

The research model is shown in Figure 2 and the hypothetical relationships between the constructs are discussed in the subsequent sections.

### 2.2. Research Hypotheses

#### 2.2.1. PU and PEOU

According to Davis et al. [16] (p. 985), PU refers to “the degree to which a person believes that using a particular system would enhance his or her job performance”, whilst PEOU denotes “the degree to which an individual believes that using a particular system would be free of physical and mental effort”. In other words, while PU reflects individuals’ perceptions concerning “the outcome of the experience”, PEOU denotes their perceptions concerning “the process leading to the final outcome” [35] (p. 104). Applying this to the context of online food shopping, we refer to PU as consumer perception that purchasing food online will enhance their shopping experience and performance. Moreover, we define PEOU as consumer perception that online food shopping will require a minimum of effort.

According to the TAM, PU is affected by PEOU. Empirical research on online shopping has found that that PEOU exerts a positive impact on PU in both developed and emerging markets [26,36,37]. That is, the easier it is for consumers to use internet connected devices or websites for purchasing products online, the more useful online shopping is perceived to be by these consumers [13,17,25,35,38]. A survey of online grocery shoppers conducted by Kurnia and Chien [39] reveals that the relationship between PEOU and PU is significant and positive. Kim and Woo [40] applied the TAM to explain the use of quick response (QR) codes for traceability systems among consumers in the emerging economy of South Korea, and they discovered that PEOU exerts a positive impact on PU. Hence, we formulated the following hypothesis.

**Hypothesis** **1.** *Consumers’ PEOU of online food purchasing will have a positive influence on their PU of online food purchasing.*

#### 2.2.2. Attitude towards Online Food Purchasing

Attitude towards a specified behavior is defined as “the degree to which a person has a favorable or unfavorable evaluation or appraisal of the behavior in question” [41] (p. 188). According to the TAM [16], both PEOU and PU are motivators of consumer attitude towards using a new technology or system. In the online shopping context, consumers will develop positive attitudes towards buying products online when they perceive that devices or tools connected to the internet are easy to operate. Prior research studies have found that consumers’ PEOU of retail websites and online shopping has a positive effect on their attitudes towards purchasing products online. PU is also an important determinant of attitude because the more useful customers perceive online shopping to be, the more favorable are their attitudes towards online shopping [13,38].

Several studies of online food shopping have confirmed the significant association between PEOU, PU, and attitude. Notably, Kurnia and Chien [39] investigated various factors influencing Australian consumers’ acceptance of online grocery shopping and found that PEOU and PU are the strongest predictors of attitude. Their findings are supported by Kim and Woo [40], who reported that PEOU and PU motivate South Korean consumers’ attitudes towards utilizing QR codes for food traceability systems. Another study in the context of emerging markets, conducted by Alagoz and Hekimoglu [4], demonstrated that PEOU and PU significantly enhanced attitude towards online food ordering among Turkish consumers. We have therefore proposed the following set of hypotheses.

**Hypothesis** **2.** *Consumers’ PEOU of online food purchasing will have a positive influence on their attitudes towards online food purchasing.*

**Hypothesis** **3.** *Consumers’ PU of online food purchasing will have a positive influence on their attitudes towards online food purchasing.*

#### 2.2.3. Intention towards Online Food Purchasing

Behavioral intention refers to “how hard people are willing to try” and “how much of an effort they are planning to exert” to perform a certain behavior [40] (p. 181). In the TAM, the intention of a user towards a new system or technology is strongly affected by their PU and attitude towards using the technology [16,23]. The relationships between these constructs have been confirmed in several studies that have examined consumer online shopping [26,35,42].

In the area of online food shopping, a qualitative study by Ramus and Asger Nielsen [2] revealed that the usefulness of online purchasing, which is demonstrated through its convenience, wide range of products, and time saving, is important among consumers when they form their intention to purchase food online. Likewise, Kim Dang et al. [10] asserted that convenience is the main motivation for online food shopping in the emerging country of Vietnam. Quevedo-Silva et al. [43] found that attitude had a positive relation with the intention to purchase food products online among Brazilian shoppers. This finding is echoed by Loketkrawee and Bhatiasevi [44] who claimed that attitude exerts a strong impact on Thai consumers’ intentions to use online grocery shopping. In addition, Kurnia and Chien [39] asserted that both PU and attitude have a positive effect on intention to buy groceries online. Therefore, the following hypotheses were developed.

**Hypothesis** **4.** *Consumers’ PU of online food purchasing will have a positive influence on their intentions towards online food purchasing.*

**Hypothesis** **5.** *Consumers’ attitude towards online food purchasing will have a positive influence on their intentions towards online food purchasing.*

#### 2.2.4. Trust

Trust can be viewed as “a set of specific beliefs dealing primarily with the integrity, benevolence, and ability of another party”, and it refers to “an expectation that others one chooses to trust will not behave opportunistically by taking advantage of the situation” [26] (pp. 54–55). Trust plays a vital role in e-commerce because online customers cannot touch, see, or check the quality of a product. Additionally, they are concerned about the safety and security when making online payment [45]. Trust in online shopping may involve trust in the computer system, security, privacy [46,47], vendors’ integrity, feedback or advice [20,35,48], and the retailers’ website [49]. Generally, trust is an important predictor of attitude towards buying online [13,27,35,50,51] and the behavioral intention of online consumers [13,48,52].

Our study focuses on website trust, which refers to a consumer’s beliefs about the security, integrity, and reliability of a retailer website, which is important for online food purchasing. Loketkrawee and Bhatiasevi [44] found that website trust is positively associated with attitude towards online grocery shopping, whilst Alagoz and Hekimoglu [4] discovered that online trust enhances attitudes towards online food ordering. Furthermore, Mortimer et al. [53] reported that trust has a positive impact on behavioral intention among infrequent online grocery customers. Authors such as Naresh et al. [54] and Keh and Shieh [55] suggested that trust in online shopping and websites plays a critical role in consumer decisions to purchase food online. Website trust was also found to be significantly positively correlated with online purchasing intention for grocery products in the emerging country context [44]. Hence, the following hypotheses are proposed.

**Hypothesis** **6.** *Consumers’ website trust will have a positive effect on their attitudes towards online food purchasing.*

**Hypothesis** **7.** *Consumers’ website trust will have a positive effect on their intentions towards online food purchasing.*

## 3. Research Methodology

### 3.1. Questionnaire and Measures

The questionnaire was designed using forward and backward translation between English and Vietnamese (Behling and Law, 2000). To ensure the validity and reliability of the measures, most of the items for measuring the constructs in this study’s research framework were adapted from validated scales in prior studies. For the purpose of the face validity of the measures, two marketing scholars and two experts in online food retailing were invited to review the questionnaire. In addition, to ensure the wordings and meanings of the items, 22 consumers were invited to voluntarily participate in a pilot test.

The final survey questionnaire comprises three sections, as follows: The introduction, demographic questions, and the items for measuring the five constructs examined. The items used in our study are indicated in Table 1. All measures are designed on a five-point Likert scale, ranging from 1 “strongly disagree” to 5 “strongly agree”.

Three items operationalizing PU sought the respondents’ perceptions on how online food purchasing saves their time, provides comparative shopping options, and increases the effectiveness of their shopping. Another three items for measuring PEOU sought the respondents’ perceptions on the ease of online food purchasing and ordering. Four items operationalizing website trust sought the respondents’ beliefs about the security, reliability, and clarity of the food retailer’s website. Three items for measuring attitude sought the respondents’ overall beliefs about online food purchasing. Finally, another three items operationalizing intention sought the respondents’ likelihood of purchasing food through websites and recommending online food shopping to their friends in the future.

### 3.2. Data Collection and Sample

Given the proliferation of the internet, online surveys have been considerably adopted by researchers [61]. An online survey was carried out on Vietnamese people who had purchased food online through websites. Data collection was undertaken in April and May, 2019. The data collection protocol ensuring research ethics was approved by the Chair of Research Committee at the Faculty of Marketing, Thuongmai University.

Given the lack of a proper sampling frame, convenience sampling was used. We first conveniently identified a total of 1000 potential respondents who were members of four online shopping groups on Facebook. After that, we sent a message to each respondent to invite them to participate in the survey. In the message, we specifically explained the academic purpose of the survey, and emphasized that collected data would be used for the study only and would not be disclosed for commercial purposes. There were 332 respondents who agreed to participate in the survey and returned their questionnaires. In order to minimize potential bias, no incentive was offered to respondents. Prior to data analysis, questionnaires with invalid responses, missing data, and outliers were removed, resulting in 319 usable responses. The sample profile is illustrated in Table 2.

The vast majority of the respondents were female, accounting for 69.3%. With respect to age, Millennials and Generation Z accounted for approximately 91% of the sample. Such age groups are relevant for studying online food purchasing because they are among the most active online food shoppers [6]. Moreover, 55.17% of respondents were single, followed by the married group with 42.63%. The participants were well-educated, 59.56% held an undergraduate degree and 31.66% held a master or doctoral degree. Most of the respondents were professionals/officers (51.72%) and students (42.95%) with rich experience in Internet usage (45.14% from 5–10 years and 39.18% from 10–20 years).

## 4. Results

### 4.1. Descriptive Statitistics and Reliability

Table 3 demonstrates the results of descriptive analysis and reliability analysis. The mean scores of the constructs ranged from 2.906 to 3.974. It is important to note that website trust received the lowest mean score (Mean = 2.906, which is below the midpoint of 3.0). This indicates a low level of trust in food retailers’ websites among Vietnamese shoppers. Reliability analysis was performed to generate Cronbach’s Alpha (α) value. According to Hair et al. [62], construct reliability is ensured when the α value is greater than 0.7. As shown in Table 3, all the constructs’ α values were greater than 0.7 (ranging from 0.783 to 0.889), indicating good internal consistency of reliability.

### 4.2. Structural Equation Modeling

Structural equation modeling (SEM) was applied to validate the research model and hypotheses. This analysis technique has been widely used in empirical studies that investigate consumer attitude and purchase intention associated with food products and online food shopping [44,63,64,65]. Following the two-stage structural equation modeling (SEM) by Anderson and Gerbing [66], a confirmatory factor analysis (CFA) was initially performed to assess the validity of the constructs and the measurement model. A structural model was then used to test the hypotheses. In SEM, the measurement and the structural models should demonstrate a good fit to the data, which can be assessed through some commonly-applied goodness-of-fit (GOF) indices [62]. They are χ^2^/df (chi-square normalized by degrees of freedom), GFI (goodness-of-fit index), CFI (comparative fit index), TLI (Tucker and Lewis index), and RMSEA (root mean square error of approximation). The model fit is considered good when χ^2^/df is less than five [67], the values of GFI, CFI, and TLI all exceed 0.90, and RMSEA is less than 0.08 [68,69]. From the available range of software packages, SPSS and AMOS (IBM, New York, NY, USA) were used to perform the data analysis.

#### 4.2.1. Measurement Model

Results of the CFA show that the GOF indices satisfied the rule of thumb when all resultant statistics were above the recommended level (χ^2^/df = 2.012, GFI = 0.929, CFI = 0.965, TLI = 0.956, RMSEA = 0.056). Hence, it can be concluded that the model had a good fit to the data. In order to address the issue of convergent validity, the items’ standardized factor loadings, composite reliability (CR), and average variance extracted (AVE) were examined. As shown in Table 4, the items’ standardized factor loading (FLs) values were all significant at the 0.001 level and exceeded 0.6. Additionally, AVE ranged from 0.509 to 0.739 and CR ranged from 0.803 to 0.894. Hence, all the constructs investigated had good convergent validity [62].

Construct discriminant validity was evaluated based on AVE and maximum shared variance (MSV) and the squared correlations coefficients of other constructs. As illustrated in Table 4, the values of AVE were higher than those of MSV, and the square root of the AVE for each construct was larger than its estimated correlation coefficients with other constructs. Moreover, bivariate correlations between the constructs were less than 0.7, hence it was hardly possible for the existence of multicollinearity [70]. All these results suggested adequate discriminant validity for all the constructs.

#### 4.2.2. Structural Model and Hypotheses Testing

**Figure 3:**
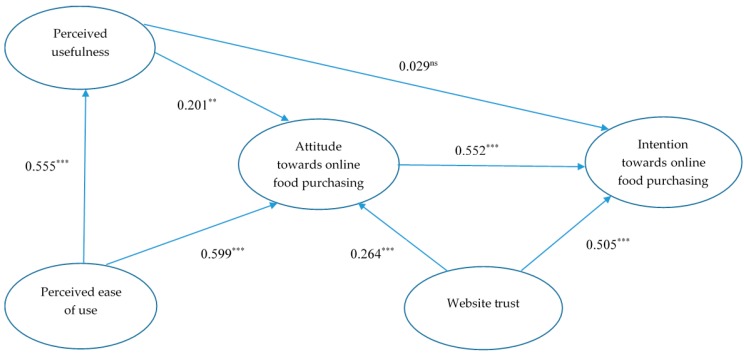
Structural model with path coefficients. Note: *** *p* < 0.001; ** *p* < 0.01; ns: nonsignificant.

Structural model was used to test the five proposed hypotheses. Figure 3 illustrates the model with the path coefficients. The resulting indices were χ^2^/df = 2.196; GFI = 0.924; CFI = 0.957; TLI = 0.948; and RMSEA = 0.061. All these fit indices suggested an adequate model fit between the empirical data and the proposed research model. The model explained 55.7% of the variance in consumer intention towards online food purchasing.

Table 5 presents the key findings from the SEM. As demonstrated in Table 5 and Figure 3, all the path relationships were statistically significant (albeit at different levels), except between PU and intention towards purchasing online food.

PEOU had a significant and positive effect on PU (β = 0.555, *p* < 0.001). Hence H_1_ is accepted. PEOU (β = 0.599, *p* < 0.001), PU (β = 0.201, *p* < 0.01), and website trust (β = 0.264, *p* < 0.001) significantly positively affected attitude towards online food purchasing, in support of H_2_, H_3_, and H_6_. Notably, among the three determinants of attitude associated with online food purchasing, PEOU exerted the greatest influence on such an attitude. Whilst attitude (β = 0.552, *p* < 0.001) and trust (β = 0.505, *p* < 0.001) were significantly positively associated with attitude, the relationship between PU and attitude was positive but insignificant (β = 0.029, *p* > 0.05). Therefore, H_4_ was rejected, whilst H_5_ and H_7_ were supported.

## 5. Discussion and Implications

This study has made an effort to explain consumer attitude and intention towards online food purchasing in the context of an emerging economy. To this end, the study developed and validated a model that combines the TAM with website trust. Whilst several previous studies removed attitude or intention from the TAM [4,26], our research model includes both of these constructs. The findings of our study have several important theoretical and managerial implications.

The results show that PEOU is a key driver of PU. This finding provides empirical support for the TAM [16,21,23] and also echoes the earlier finding by Kurnia and Chien [39]. It suggests that improving PEOU that reduces consumers’ physical and mental effort will enhance their beliefs about the usefulness and effectiveness of online food purchasing. In addition, PEOU has the greatest contribution to the formation of attitude towards purchasing food online. That is, online food consumers’ attitudes are strongly determined by how easy it is to order and purchase food through retailers’ websites. Such online shoppers tend to be convenience-oriented consumers who want shopping to be made easy, simple, and quick [71]. Therefore, online food retailers must endeavor to design websites that are easy to interact with and simple to use, which will reduce consumers’ energy and effort associated with their purchases of online food products.

The finding also reveals that PU strongly affects attitude towards online food purchasing, but PU is not a significant predictor of consumer intention to purchase food products online. That is, in the context of online food shopping in Vietnam, PU influences intention indirectly via attitude. This finding is partly inconsistent with the Australian study by Kurnia and Chien, who found that online grocery shoppers’ PU affects their intentions directly and indirectly via attitude [39]. However, our study’s finding is in line with prior research by Loketkrawee and Bhatiasevi, who asserted that PU leads to attitude, which in turn positively influences intention among Thai online food buyers [44]. It should also be mentioned that several studies using the TAM to explain online food shopping behavior have not examined the relationship between PU and behavioral intention [4,40]. Given that PU has a strong effect on attitude towards online food purchasing, food e-retailers should use communication programs emphasizing that online food shopping is time-saving, convenient, and effective and that it would improve consumers’ shopping performance and experience. Such programs should be developed and implemented jointly between online grocery retailers and relevant organizations such as the Association of Vietnam Retailers and the Vietnam Electronic Industries Association.

Another key finding concerns the role of trust in online food shopping. Although the respondents demonstrated a low level of trust in online food retailers’ websites, website trust was found to influence intention directly and indirectly via attitude towards online food purchasing. This finding extends that of Loketkrawee and Bhatiasevi [44], who only confirmed the influence of website trust on attitudes towards buying grocery products online. In addition, this finding and the aforementioned results together suggest that website trust plays a more important role in forming consumer intention than PU. In the context of online shopping, given consumer’s inability to touch or smell food products and their difficulty in judging the quality of the products, food retailers’ websites should effectively present detailed and honest information about food products (e.g., ingredients, nutrients, manufacturers, country of origin, etc.) and the conditions of purchasing/ordering. In addition, customer testimonials emphasizing product quality and website quality should be put on the websites to build up customer trust and relationship. Retailers and website managers should make every effort to enhance the reliability and security of their websites as well as protect consumer privacy. Privacy policies should be clearly communicated to shoppers. Such efforts should be echoed by policy makers, who should strengthen legislation that protects consumers’ privacy and prevents financial risks in online shopping. The previously mentioned measures altogether will improve consumers’ website trust, which, in turn, enhances their attitudes and intentions towards online food purchasing.

## 6. Conclusions and Future Research

Although online shopping has been extensively studied, this study is among the first of its kind in providing empirical evidence pertaining to the effects of PU, PEOU, and trust on attitude and behavioral intention among online food shoppers in Vietnam, which is an important emerging market economy in the Southeast Asia. SEM results confirm the integral roles of PU, PEOU, and trust in enhancing the attitude towards online food purchasing. Among these drivers, PEOU exerts the greatest influence on attitude. In addition, attitude and trust are powerful drivers of intention towards purchasing food online. The model developed and validated in our study can serve as a framework for evaluating online food shopping in other research contexts. This study’s findings also have several important managerial implications for developing strategies to encourage the purchase of food products online in emerging countries like Vietnam. Ideally, such strategies should be developed and implemented jointly by key stakeholders, such as online food retailers, website developers and managers, associations, and policy makers.

This study has several limitations that can be addressed in future research. It should be noted that actual behavior was not included in the research model. Whilst this is in line with several prior studies, future research is advised to incorporate the actual purchase of online food shoppers. In this regard, a longitudinal study can be performed. Future studies should also extend our research model by including factors relating to enjoyment, prior experiences, or perceived risks. Moreover, the non-probability convenience sampling that was used limits the representativeness of our sample, which includes a great proportion of female and highly educated shoppers, as well as those aged under 40. Hence, future studies should seek to apply probability sampling techniques to obtain data from a more representative sample consisting of a wider range of respondents. Another possible way to control the representativeness of the sample is to combine the currently used survey method with experiments. Such a mixed method will also strengthen the rigor of the study and the significance of its findings. Additionally, given that this study examined food products in general, future research could focus on certain food categories. Finally, it would be desirable to conduct comparative studies that identify and explain the differences in online food shopping between groups of consumers with different demographic, geographic, and cultural characteristics.

## Figures and Tables

**Table 1:** Variables and items.

Variables and Items	References Sources
Perceived usefulness (PU)	
Online food purchasing enables me to save my time.	[25]
Using the website for online food shopping makes it more effective to do my shopping.	[16,25]
Using the website for online food shopping facilitates comparative shopping.	[56]
Perceived ease of use (PEOU)	
Learning to operate online food shopping is easy for me.	[25,56]
I find it easy to become skilled at purchasing food online.	[56,57]
It is easy to order food online.	[52]
Website trust (TRU)	
Online food products in the website are reliable.	[58]
I believe in the information about food products that the website provides.	[19]
The conditions of purchasing food products are clearly indicated in the website.	[58]
The website secures customer privacy.	[58]
Attitude towards online food purchasing (ATT)	
Purchasing food through the website a good idea	[25,56]
Purchasing food through the website is a wise idea.	[56]
I like to purchase food through the website.	[56]
Intention towards online food purchasing (INT)	
I intend to use the website for food purchasing shortly.	[25,59]
I predict I will regularly use the website for food purchasing in the future.	[25,59]
I intend to recommend online food shopping to my friends.	[60]

**Table 2:** Sample profile.

Demographic Variables	Respondents	
	Frequency	%
Gender		
Female	221	69.30
Male	98	30.70
Age (years)		
18–29	184	57.68
30–39	106	33.23
40–49	25	7.84
50+	4	1.25
Marital status		
Single/never married	176	55.17
Married	136	42.63
Divorced	5	1.57
Widowed	2	0.63
Education level		
High school and below	28	8.78
Undergraduate	190	59.56
Postgraduate	101	31.66
Occupation		
Students	137	42.95
Professionals/officers	165	51.72
Self-employed	8	2.51
Housewife	3	0.94
Others	6	1.88
Experience in Internet usage		
<5 years	32	10.03
5–10 years	144	45.14
>10 years	143	44.83

Note: *n* = 319.

**Table 3:** Descriptive statistics and reliability.

Constructs	Mean	SD	α
Perceived usefulness (PU)	3.974	0.898	0.805
Perceived ease of use (PEOU)	3.919	0.905	0.855
Website trust	2.906	0.703	0.783
Attitude towards online food purchasing	3.902	0.911	0.889
Intention towards online food purchasing	3.280	1.001	0.880

Note: SD: Standard deviation; α: Cronbach’s Alpha values.

**Table 4:** Convergent validity and discriminant validity.

Construct	FLs	CR	AVE	MSV	PU	PEOU	TRU	ATT	INT
PU	0.72–0.83	0.808	0.585	0.309	**0.765**				
PEOU	0.78–0.84	0.857	0.666	0.483	0.556	**0.816**			
TRU	0.60–0.82	0.803	0.509	0.304	0.125	0.322	**0.713**		
ATT	0.77–0.91	0.894	0.739	0.483	0.505	0.695	0.352	**0.859**	
INT	0.83–0.86	0.880	0.709	0.448	0.332	0.536	0.551	0.669	**0.842**

Note: Diagonal value (bold) indicates the square root of average variance extracted (AVE) of construct.

**Table 5:** SEM results and hypotheses testing.

Hypotheses			β	S.E.	*t*-Value	*p*-Value	Findings
H_1_: PEOU	→	PU	0.555	0.070	7.969	***	Supported
H_2_: PEOU	→	ATT	0.599	0.073	8.176	***	Supported
H_3_: PU	→	ATT	0.201	0.071	2.837	**	Supported
H_4_: PU	→	INT	0.029	0.067	0.436	0.663	Not supported
H_5_: ATT	→	INT	0.552	0.066	8.414	***	Supported
H_6_: TRU	→	ATT	0.264	0.070	3.777	***	Supported
H_7_: TRU	→	INT	0.505	0.076	6.646	***	Supported

Note: *** *p* < 0.001; ** *p* < 0.01; β: standardized path coefficients; S.E. Standard error; “→”indicates the direction of the hypothesis.

## References

[1] Nielsen (2018). Nielsen Connected Commerce Report Examines Consumers’ Online Purchasing Behavior across 64 Countries.

[2] Ramus K., Asger Nielsen N. (2005). Online grocery retailing: What do consumers think?. Internet Res..

[3] Hartono E., Holsapple C.W., Kim K.Y., Na K.S., Simpson J.T. (2014). Measuring perceived security in B2C electronic commerce website usage: A respecification and validation. Decis. Support Syst..

[4] Alagoz S.H., Hekimoglu H. (2012). A study on tam: Analysis of customer attitudes in online food ordering system. Procedia Soc. Behav. Sci..

[5] Statista (2017). Facebook Usage Penetration in Vietnam from 2015 to 2022.

[6] Nielsen (2015). The Future of Grocery: E-Commerce, Digital Technology and Changing Shopping Preferences around the World.

[7] EVBN (2018). E-Commerce Industry in Vietnam.

[8] PwC (2018). The Future of Asean: Vietnam Perspective.

[9] Chung C., Muk A. (2017). Online Shoppers’ Social Media Usage and Shopping Behavior.

[10] Dang A.K., Tran B.X., Nguyen C.T., Le H.T., Do H.T., Nguyen H.D., Nguyen L.H., Nguyen T.H., Mai H.T., Tran T.D. (2018). Consumer preference and attitude regarding online food products in hanoi, vietnam. Int. J. Environ. Res. Public Health.

[11] Van der Heijden H., Verhagen T., Creemers M. (2003). Understanding online purchase intentions: Contributions from technology and trust perspectives. Eur. J. Inf. Syst..

[12] Evans C., Hackney R., Rauniar R., Rawski G., Yang J., Johnson B. (2014). Technology acceptance model (tam) and social media usage: An empirical study on facebook. J. Enterp. Inf. Manag..

[13] Çelik H.E., Yilmaz V. (2011). Extending the technology acceptance model for adoption of e-shopping by consumers in turkey. J. Electron. Commer. Res..

[14] Gefen D. (2000). E-commerce: The role of familiarity and trust. Omega.

[15] Changchit C., Cutshall R., Lonkani R., Pholwan K., Pongwiritthon R. (2018). Determinants of online shopping influencing thai consumer’s buying choices. J. Internet Commer..

[16] Davis F.D., Bagozzi R.P., Warshaw P.R. (1989). User acceptance of computer technology: A comparison of two theoretical models. Manag. Sci..

[17] Sondakh J.J. (2017). Behavioral intention to use e-tax service system: An application of technology acceptance model. Eur. Res. Stud..

[18] Tuteja G., Gupta S., Garg V. (2016). Consumer trust in internet shopping: An empirical investigation. Paradigm.

[19] Bilgihan A. (2016). Gen y customer loyalty in online shopping: An integrated model of trust, user experience and branding. Comput. Hum. Behav..

[20] Xiao Z., Zhang J., Li D., Chen C. (2015). Trust in online food purchase behavior: An exploration in food safety problem for produce e-retailers. Adv. J. Food Sci. Technol..

[21] Davis F.D. (1986). A Technology Acceptance Model for Empirically Testing New End-User Information Systems: Theory and Results.

[22] Fishbein M., Ajzen I. (1975). Belief, Attitude, Intention, and Behavior: An Introduction to Theory and Research.

[23] Davis F.D. (1989). Perceived usefulness, perceived ease of use, and user acceptance of information technology. MIS Q..

[24] Marangunić N., Granić A. (2015). Technology acceptance model: A literature review from 1986 to 2013. Univers. Access Inf. Soc..

[25] Bauerová R., Klepek M. (2018). Technology acceptance as a determinant of online grocery shopping adoption. Acta Univ. Agric. Silvic. Mendel. Brun..

[26] Gefen D., Karahanna E., Straub D.W. (2003). Trust and tam in online shopping: An integrated model. MIS Q..

[27] Hassanein K., Head M. (2007). Manipulating perceived social presence through the web interface and its impact on attitude towards online shopping. Int. J. Hum. Comput. Stud..

[28] King W.R., He J. (2006). A meta-analysis of the technology acceptance model. Inf. Manag..

[29] Koufaris M. (2002). Applying the technology acceptance model and flow theory to online consumer behavior. Inf. Syst. Res..

[30] Michelle Bobbitt L., Dabholkar P.A. (2001). Integrating attitudinal theories to understand and predict use of technology-based self-service: The internet as an illustration. Int. J. Serv. Ind. Manag..

[31] Pavlou P.A., Fygenson M. (2006). Understanding and predicting electronic commerce adoption: An extension of the theory of planned behavior. MIS Q..

[32] Toufaily E., Souiden N., Ladhari R. (2013). Consumer trust toward retail websites: Comparison between pure click and click-and-brick retailers. J. Retail. Consum. Serv..

[33] Nguyen H.V., Nguyen N., Nguyen B.K., Lobo A., Vu P.A. (2019). Organic food purchases in an emerging market: The influence of consumers’ personal factors and green marketing practices of food stores. Int. J. Environ. Res. Public Health.

[34] Nguyen-Viet H., Tuyet-Hanh T.T., Unger F., Dang-Xuan S., Grace D. (2017). Food safety in vietnam: Where we are at and what we can learn from international experiences. Infect. Dis. Poverty.

[35] Monsuwé T.P.Y., Dellaert B.G., De Ruyter K. (2004). What drives consumers to shop online? A literature review. Int. J. Serv. Ind. Manag..

[36] Gefen D., Straub D.W. (2000). The relative importance of perceived ease of use in is adoption: A study of e-commerce adoption. J. Assoc. Inf. Syst..

[37] Kim J.B. (2012). An empirical study on consumer first purchase intention in online shopping: Integrating initial trust and tam. Electron. Commer. Res..

[38] Lim W.M., Ting D.H. (2012). E-shopping: An analysis of the technology acceptance model. Mod. Appl. Sci..

[39] Kurnia S., Chien J. The acceptance of the online grocery shopping. Proceedings of the 16th Bled Electronic Commerce Conference.

[40] Kim Y.G., Woo E. (2016). Consumer acceptance of a quick response (qr) code for the food traceability system: Application of an extended technology acceptance model (tam). Food Res. Int..

[41] Ajzen I. (1991). The theory of planned behavior. Organ. Behav. Hum. Decis. Process..

[42] Chang M.K., Cheung W., Lai V.S. (2005). Literature derived reference models for the adoption of online shopping. Inf. Manag..

[43] Quevedo-Silva F., Freire O., Lima-Filho D., Brandão M., Isabella G., Moreira L. (2016). Intentions to purchase food through the internet: Developing and testing a model. Br. Food J..

[44] Loketkrawee P., Bhatiasevi V. (2018). Elucidating the behavior of consumers toward online grocery shopping: The role of shopping orientation. J. Internet Commer..

[45] Lee M.K., Turban E. (2001). A trust model for consumer internet shopping. Int. J. Electron. Commer..

[46] Kim S., Williams R., Lee Y. (2004). Attitude toward online shopping and retail website quality: A comparison of us and korean consumers. J. Int. Consum. Mark..

[47] Ranganathan C., Ganapathy S. (2002). Key dimensions of business-to-consumer web sites. Inf. Manag..

[48] Gefen D., Benbasat I., Pavlou P. (2008). A research agenda for trust in online environments. J. Manag. Inf. Syst..

[49] Ogonowski A., Montandon A., Botha E., Reyneke M. (2014). Should new online stores invest in social presence elements? The effect of social presence on initial trust formation. J. Retail. Consum. Serv..

[50] Ha S., Stoel L. (2009). Consumer e-shopping acceptance: Antecedents in a technology acceptance model. J. Bus. Res..

[51] Jarvenpaa S.L., Tractinsky N., Vitale M. (2000). Consumer trust in an internet store. Inf. Technol..

[52] Butt I., Tabassam S., Chaudhry N.G., Nusair K. (2016). Using technology acceptance model to study adoption of online shopping in an emerging economy. J. Internet Bank..

[53] Mortimer G., Fazal e Hasan S., Andrews L., Martin J. (2016). Online grocery shopping: The impact of shopping frequency on perceived risk. Int. Rev. Retail Distrib. Consum. Res..

[54] Naresh N., Pai A., Prabhu N., Kumar M. (2015). Online shopping patterns amidst students with respect to food products. Int. J. Manag. Soc. Sci. Res..

[55] Keh H.T., Shieh E. (2001). Online grocery retailing: Success factors and potential pitfalls. Bus. Horiz..

[56] Lin H.F. (2007). Predicting consumer intentions to shop online: An empirical test of competing theories. Electron. Commer. Res. Appl..

[57] Park S.Y. (2009). An analysis of the technology acceptance model in understanding university students’ behavioral intention to use e-learning. Educ. Technol. Soc..

[58] Chen S.H., Lee K.P. (2008). The role of personality traits and perceived values in persuasion: An elaboration likelihood model perspective on online shopping. Soc. Behav. Personal. Int. J..

[59] Van der Heijden H. (2004). User acceptance of hedonic information systems. MIS Q..

[60] Yang H.H., Su C.H. (2017). Learner behaviour in a mooc practice-oriented course: In empirical study integrating tam and tpb. Int. Rev. Res. Open Distrib. Learn..

[61] Bhattacherjee A. (2001). Understanding information systems continuance: An expectation-confirmation model. MIS Q..

[62] Hair J., Black W., Babin B., Anderson R. (2010). Multivariate Data Analysis.

[63] Pérez-Villarreal H.H., Martínez-Ruiz M.P., Izquierdo-Yusta A. (2019). Testing model of purchase intention for fast food in mexico: How do consumers react to food values, positive anticipated emotions, attitude toward the brand, and attitude toward eating hamburgers?. Foods.

[64] Suzuki T., Oishi T., Kurokura H., Yagi N. (2019). Which aspects of food value promote consumer purchase intent after a disaster? A case study of salmon products in disaster-affected areas of the great east japan earthquake. Foods.

[65] Pham T.H., Nguyen T.N., Phan T.T.H., Nguyen N.T. (2019). Evaluating the purchase behaviour of organic food by young consumers in an emerging market economy. J. Strateg. Mark..

[66] Anderson J.C., Gerbing D.W. (1988). Structural equation modeling in practice: A review and recommended two-step approach. Psychol. Bull..

[67] Bentler P.M. (1995). Eqs Structural Equations Program Manual.

[68] Scott J.E. (1995). The measurement of information systems effectiveness: Evaluating a measuring instrument. ACM SIGMIS Database.

[69] Henry J.W., Stone R.W. (1994). A structural equation model of end-user satisfaction with a computer-based medical information system. Inf. Resour. Manag. J..

[70] Grewal R., Cote J.A., Baumgartner H. (2004). Multicollinearity and measurement error in structural equation models: Implications for theory testing. Mark. Sci..

[71] Handa M., Gupta N. (2014). A study of the relationship between shopping orientation and online shopping behavior among indian youth. J. Internet Commer..

